# Optimizing Nutrition Assessment to Create Better Outcomes in Lung Transplant Recipients: A Review of Current Practices

**DOI:** 10.3390/nu11122884

**Published:** 2019-11-27

**Authors:** Mara Weber Gulling, Monica Schaefer, Laura Bishop-Simo, Brian C. Keller

**Affiliations:** 1 Nutrition Services, The Ohio State University Wexner Medical Center, Columbus, OH 43210, USA; Mara.Weber@osumc.edu (M.W.G.); Monica.Schaefer@osumc.edu (M.S.); Laura.Bishop-Simo@osumc.edu (L.B.-S.); 2 Division of Pulmonary, Critical Care & Sleep Medicine, The Ohio State University College of Medicine, Columbus, OH 43210, USA

**Keywords:** lung transplantation, body mass index, nutrition, body composition, lean body mass, muscle mass, leptin, sarcopenia, creatinine-height index

## Abstract

Lung transplantation offers patients with end-stage lung disease an opportunity for a better quality of life, but with limited organ availability it is paramount that selected patients have the best opportunity for successful outcomes. Nutrition plays a central role in post-surgical outcomes and, historically, body mass index (BMI) has been used as the de facto method of assessing a lung transplant candidate’s nutritional status. Here, we review the historical origins of BMI in lung transplantation, summarize the current BMI literature, and review studies of alternative/complementary body composition assessment tools, including lean psoas area, creatinine-height index, leptin, and dual x-ray absorptiometry. These body composition measures quantify lean body mass versus fat mass and may provide a more comprehensive analysis of a patient’s nutritional state than BMI alone.

Institutional lung transplant patient selection committees are faced with a daunting task: to accurately and expediently identify and predict candidates with a high likelihood of success following lung transplantation. The selection process involves a rigorous and comprehensive evaluation of the potential candidate’s medical and surgical comorbidities, physical fitness and potential for improvement, and social and financial support. Also included in this assessment—though sometimes overlooked in its importance, particularly in the setting of critically ill patients—is nutritional status. In this review, we discuss the pre-transplant evaluation of nutritional status in patients with end-stage lung disease which, historically, has utilized the body mass index (BMI), and we discuss recent developments that may enhance our future ability to more finely characterize a candidate’s preoperative nutritional status with the aim of better prognosticating outcomes after lung transplantation.

## 1. Lung Transplant Candidate Selection

Lung transplantation is the definitive treatment for end-stage lung disease and offers these patients the opportunity for better quality of life. Lung transplant rates continue to increase, with 2345 lung transplants performed in the USA in 2016. Yet, at the end of the year, 1395 candidates remained on the waiting list [1]. Despite the rise in numbers of transplants performed, survival outcomes after lung transplantation remain far lower than those of other solid organs, with a median survival of approximately 6 years [2]. Given the limited number of organs available and, in contrast, the large number of individuals awaiting transplantation, it is paramount that patients selected for transplant have the best potential for successful outcomes.

The Ohio State University Transplant Center follows a comprehensive lung transplant patient evaluation process based on the recommendations of The International Society for Heart & Lung Transplantation (ISHLT) [3]. Patients undergo an extensive review of their medical, surgical, family, social, medication, and allergy history. Pulmonary tests include chest radiography, chest computed tomography, quantitative lung perfusion, spirometry with lung volumes and diffusion capacity for carbon monoxide, 6-min walk test, and room air arterial blood gas testing. Cardiac and gastrointestinal testing includes echocardiogram and right/left heart catheterization and gastric emptying, esophageal pH testing, and esophageal manometry, respectively. Patients are screened for infectious diseases via survey and bloodwork, and health maintenance screenings are updated. Psychosocial evaluations are performed by social workers and/or psychologists/psychiatrists, and all potential candidates undergo a nutrition evaluation by a registered dietitian. Finally, a financial assessment, including insurance, is completed before the patient is brought to the patient selection committee.

Despite best efforts to select the most suitable candidates, there are areas for improvement. As described above, there are many facets to lung transplant candidate evaluation, including nutrition status. Within the realm of nutrition is a patient’s body composition and state of nourishment. Use of historical as well as novel tools can facilitate a more objective assessment of a patient’s body composition and nutrition status and potentially identify targets for early or aggressive intervention. Given that this area of medical science is young and developing, expert opinion has been the basis of much of the criteria available [4].

## 2. Nutrition Evaluation

When a patient is referred to our center, a preliminary nutrition screening is performed over the phone by a pre-transplant nurse coordinator who collects the patient’s height and weight for the calculation of BMI. For the purpose of this article and the studies reviewed, BMI is calculated as weight (kg) divided by height^2^ (m^2^). Weight ranges are based on the World Health Organization (WHO) classification scheme: underweight (<18.5 kg/m^2^); normal weight (18.5–24.9 kg/m^2^); overweight (25–29.9 kg/m^2^); and obese (>30 kg/m^2^) [5].

A transplant pulmonologist completes an initial in-person assessment, and should the patient be deemed appropriate for full evaluation, additional consults and tests are arranged [6], including a full nutrition assessment with the transplant dietitian. The full nutrition evaluation has evolved and involves reviewing the history of present illness; past medical, surgical, and social histories; medication review; activity level, including review of 6-min walk test results; and diet history, including current diet, eating behaviors, current symptoms affecting oral intake, diabetes history, weight history, previous weight gain/loss attempts, food allergies, current and past use of oral nutrition supplement products, and a Nutrition Focused Physical Exam (hand grip currently not performed) [6]. Patients are then assigned a malnutrition diagnosis based on the 2012 Academy of Nutrition and Dietetics/American Society for Parental and Enteral Nutrition (AND/ASPEN) Guidelines [7], and an assessment is made of relative and absolute contraindications based on transplant center policy, transplant nutrition-related experience, and nutrition diagnosis. Finally, an individualized nutrition plan of care/goals, based on the patient and their readiness for change, resources available, severity of illness, and other factors, is developed. Additional support from other nutrition providers is utilized when possible, including comprehensive weight management services, nutrition care associated with pulmonary rehabilitation, and diabetes management, as well as other appropriate services dependent on patient needs and willingness/ability to participate.

Following completion of the pre-transplant assessment and testing, patients are presented at the Lung Transplant Patient Selection Committee for multidisciplinary team review. Relative and absolute contraindications are reviewed, including those that are pertinent from a nutrition perspective. Generally, much like the ISHLT Pulmonary Council, our center considers an absolute contraindication to lung transplant to be class II or III obesity (BMI ≥ 35 kg/m^2^) [3]; obese individuals are, however, evaluated on a case by case basis and offered support to achieve a more desirable weight. Relative contraindications associated with nutrition care include class I obesity (BMI > 30–34.9 kg/m^2^), underweight BMI < 17 kg/m^2^, cachexia, and malnutrition, as well as uncontrolled diabetes and osteoporosis [3]. For the purposes of this review, we focus on the use of BMI and malnutrition in the lung transplant evaluation and subsequent outcomes (Table 1).

## 3. BMI-Based Nutrition Assessment

The nutritional needs of the lung transplant population are immensely varied; individualized nutrition prescriptions are necessary to best serve each person, which can create challenges when studying this population. Broadly, transplants are provided for patients with four categories of lung disease: obstructive, suppurative, restrictive, and vascular [4]. As a result, transplant recipients and their nutritional needs can vary from young underweight cystic fibrosis (CF) patients to the older chronic obstructive pulmonary disease and pulmonary fibrosis patients that may be obese. Across the board, patients’ candidacy still relies in part on weight status, usually in the form of BMI.

Interestingly, BMI itself dates back to the 19th century, when Belgian Adolphe Quetelet suggested the premise that “the transverse growth of man is less than the vertical” [qtd. in 13] and thus derived the equation weight (kg) divided by height (m) squared. It was not until much later that Ancel Keys coined the term “body mass index” with evidence to support the theory [13]. While BMI has long been the standard method of assessing nutrition status in potential candidates because of its ease and low cost, it was not always so. In fact, in the inaugural 1998 guidelines for selection of lung transplant candidates, ideal body weight (IBW) was recommended as the measure of nutritional status. Patients with IBW <70% or >130% were required to gain or lose weight, respectively, in order to move forward with transplant [14].

Historically, underweight and obese lung transplant recipients have been linked with poor post-surgical outcomes, though studies have been conflicting [12]. The utility of BMI in predicting lung transplant outcomes came to light with Plöchl’s report of increased intensive care unit (ICU) mortality in lung transplant recipients whose BMI was in the lowest quartile [8], though no difference was observed in ICU length of stay (LOS). Following this, studies by Madill et al. and Kanasky et al. in the early 2000s were the first to demonstrate longer term adverse outcomes in lung transplantation at the extremes of nutrition status as measured by BMI [5,10]. In the first study, recipients with BMI <17 kg/m^2^ or >25 kg/m^2^ were associated with higher risk of death in the first 90 days compared to those with BMI between 20–25 kg/m^2^ [10]. Recipients with BMI >27 kg/m^2^ were even more at risk, with a 5-fold higher odds ratio of death compared to the reference group. In a single-center retrospective review of 85 patients, Kanasky et al. found that patients classified as obese (BMI ≥ 30 kg/m^2^) prior to transplantation had markedly shorter post-transplant survival times (40% survival at 20 months for BMI ≥ 30 kg/m^2^ versus nearly 70% survival at 50 months for BMI < 30 kg/m^2^). Underweight patients (BMI < 18.5 kg/m^2^) had better survival in the first 50 months post-transplant compared to normal (18.5–24.9 kg/m^2^) or overweight (25–29.9 kg/m^2^) recipients, with a late marked decline thereafter [5], a departure from Plöchl’s findings. In contrast to Madill’s earlier study, there was no difference in survival between normal and overweight recipients.

Based on these studies, BMI was eventually incorporated as a component of the lung allocation score, a method of prioritizing lung transplant candidates, introduced in 2005 [15], and BMI > 30 kg/m^2^ was included as a relative contraindication to transplant in the 2006 update of lung transplant candidate selection guidelines [16]. In the most recent 2014 update to the guidelines, class II or III obesity (BMI ≥ 35 kg/m^2^) is now considered an absolute contraindication to lung transplant, while class I obesity (BMI 30–34.9 kg/m^2^) remains a relative contraindication [3].

However, as newer methods for measuring body composition are developed, BMI’s role as the sole measure of nutrition status in lung transplant candidates is now being questioned. Inconsistencies among the aforementioned studies may be due to BMI’s inability to discriminate different body compositions (adipose tissue mass and muscle mass). Consequently, the use of BMI alone can place patients in a gray area when determining their transplant candidacy, especially for those who are asked to gain or lose weight prior to transplant listing, a difficult task in patients with end-stage lung disease. Therefore, use of alternative and complementary modalities to BMI may better delineate a candidate’s nutrition status.

## 4. Creating Better Outcomes through Use of BMI Alternatives

Body composition can be computed a number of different ways, including, but not limited to, computed tomography (CT), magnetic resonance imaging (MRI), dual x-ray absorptiometry (DXA), and bioimpedance analysis (BIA). Both CT and MRI are considered the gold standards for estimating muscle mass in research [17]. Unfortunately, there are drawbacks (e.g., exposure to radiation, higher cost, and availability of equipment) to using these gold standard methods routinely in the clinic that may contribute to the continued use of BMI as a means of assessing candidates [18]. While BMI is most frequently used in the assessment of lung transplant candidates, our notion that body composition may produce better outcomes is not considered novel. Several studies, including those highlighted below, analyzed body composition through CT, DXA, as well as creatinine-height index (CHI), and measured leptin levels.

### 4.1. Lean Psoas Area

Sarcopenia is defined by decreased muscle mass and either low peripheral muscle strength or function and becomes more prevalent with aging [18]. Assessment of muscle tone therefore becomes that much more essential, particularly in lung transplant candidates, a population that has steadily increased in age [19]. Moreover, sarcopenia affects people of all weight classes. Core muscle size, estimated by lean psoas area (LPA) using CT scans, was evaluated as a predictor of postoperative outcomes in lung transplant recipients. LPA, not BMI, was associated with shorter duration of mechanical ventilation, reduced need for tracheostomy, and shorter ICU LOS [12]. Six-minute walk distance increased with increasing LPA and decreased with increasing BMI. Sarcopenia (measured as low LPA) was present in one-third of normal weight and one-fourth of overweight patients. On the other hand, half of the underweight population had normal or high LPA. While further studies are needed, it is likely that transplant outcomes will be less favorable in sarcopenic patients with normal or elevated BMI due to BMI’s inability to account for body composition.

### 4.2. Leptin and DXA

In contrast to the aforementioned BMI studies, Singer et al. found that class I obesity (BMI 30–34.9) was not associated with one-year mortality after lung transplant [11]. However, using DXA and measured leptin levels, the authors identified a linkage between body composition and survival. Leptin, a satiety hormone produced by adipose cells, is required for energy balance [20]. Mutations in *LEP*, the gene encoding leptin, led to altered metabolism and the development of obesity [20]. Compared to BMI, leptin levels correlate more strongly with percent body fat [21]. In lung transplant recipients, higher preoperative plasma leptin levels were associated with increased one-year mortality in patients not requiring intraoperative cardiopulmonary bypass support. Body composition analysis with DXA identified obesity in 51% of patients with a normal BMI. Conversely, sarcopenia was noted in 46% of patients by DXA, whereas only 5% of patients were classified as underweight by BMI alone. This data suggests that nutrition analysis by BMI alone may be insufficient. For patients classified as underweight based on BMI, body composition analysis may have mitigated the need for pre-transplant weight gain and, thus, delay in transplant listing. The reverse is also true; patients with sarcopenic obesity could be educated on muscle mass maintenance rather than strict weight loss, which, in turn, may improve outcomes such as shortening post-transplant hospital LOS [22].

### 4.3. Creatinine-Height Index

The creatinine-height index (CHI) was developed in the 1970s as a measure of protein nutrition and lean muscle mass [23] and is calculated as:

$$\mathrm{CHI}=\frac{24\;h\;\mathrm{urinary}\;\mathrm{creatinine}\;\mathrm{of}\;\mathrm{subject}}{\mathrm{expected}\;24\;h\;\mathrm{urinary}\;\mathrm{creatinine}\;\mathrm{of}\;\mathrm{person}\;\mathrm{of}\;\mathrm{same}\;\mathrm{height}\;\mathrm{and}\;\mathrm{sex}}\;\times \;100.$$

Comparing CHI and percent ideal body weight (IBW), Schwebel et al. noted that nutritional depletion was prevalent in lung transplant candidates and was a risk factor for higher mortality. In this study, 72% had some form of nutritional depletion, which included patients with weights <90% and ≥90% of IBW. Furthermore, lower 6-min walk distance and more severe hypoxemia were observed in individuals with a higher percent IBW but poorer lean body mass as estimated by CHI, even more so than those with low weight and low lean body mass. Therefore, lean body mass depletion is not exclusively tied to decreased body weight. This research supports the use of supplemental measures, like CHI, to more precisely assess nutritional status and ultimately predict post-transplant outcomes. The authors hypothesized that improving nutritional status pre-transplant might improve body composition quality and therefore reduce ICU LOS and overall costs [9].

## 5. Optimizing Nutrition in Lung Transplant Candidates

### 5.1. Outcomes in Relation to Nutritional State

With the understanding that nutrition, with time, can improve patient health before, during, and after transplantation, the role of the dietitian at each stage is essential. Unfortunately, disease states can progress quickly, and sometimes a patient’s nutrition status may be less than ideal prior to surgery despite nutritional intervention. Following transplant, similar issues may be seen. Complications during the perioperative period or longer term can have detrimental effects on patient health. In the acute perioperative phase, nutrition’s role includes promoting wound healing, preventing infection, and meeting a patient’s macronutrient needs given their catabolic state. Further beyond transplant, nutrition’s role shifts but continues to be paramount, assisting in healthy weight maintenance, aiding in blood glucose control, preventing chronic metabolic diseases, and reducing potential complications like graft rejection [24].

For those diagnosed with malnutrition, addressing weight loss and loss of lean body mass is imperative. A potential linkage between the degree of malnutrition and airflow obstruction has been reported [25]. Loss of lean body mass has been associated with a greater loss in lung function, reduced distance on 6-min walk test, and worsened hypoxemia [9,26]. Additionally, these patients are likelier to experience prolonged ICU LOS, days on mechanical ventilation, and increased mortality [8,10,12,27]. Similarly, pre-transplant serum prealbumin, a marker of both inflammation and nutrition status, has been linked to outcomes in lung transplant recipients, with low prealbumin levels (<18 g/dL) associated with a threefold higher risk of death than levels above 18 g/dL [27].

### 5.2. Weight Gains and Losses

For patients at the upper end or just above a program’s maximally acceptable BMI, weight loss is likely to be recommended prior to transplant listing. Weight loss improves survival and perioperative morbidity and leads to reduced ICU LOS and duration of mechanical ventilation [28]. As previously mentioned, obese patients are at higher risk for complications, however, strict weight loss may not guarantee better outcomes, especially if it means a loss of muscle mass [11]. Rather, emphasis on healthful weight loss (loss of fat mass rather than muscle mass, an endeavor that takes a great deal of time, which may be limiting in lung transplant candidates) would prove to be more beneficial regarding post-transplant outcomes.

In the immediate postoperative period, patients experience a decline in weight and BMI despite the best efforts of dietary staff to improve their nutritional state pre-transplant. The postoperative catabolic state and potential intestinal absorption concerns following the initiation of anti-rejection medications may play an important role in this weight loss, such that oral intake alone may be insufficient in meeting nutritional needs, especially in patients severely malnourished prior to transplant [29]. This is important because postoperative malnutrition impacts morbidity and mortality through effects on immune cell and skeletal muscle function and impaired wound healing [30], leading to a 3-fold increased risk of nosocomial infection [29].

Beyond the immediate postoperative period, a median weight gain of 10% is anticipated [31]. While not an exhaustive list, weight gain after transplant may be a result of corticosteroid use, increased leptin levels, decreased resting energy expenditure, and reduced production of cachexia-associated cytokines [31]. While steroid use in transplant patients may contribute to weight gain, due to dose tapering, this weight gain may not contribute as much as anticipated [31,32]. The most substantial weight gain occurs during the first year, and patients with successful early post-transplant weight gain demonstrate better survival [31]. CF patients and younger patients also tend to gain more weight [31,33]. The reasons for this are still not clear, but may be due to better early lung allograft function, fewer pro-inflammatory cytokines, decreased work of breathing, or enhanced immunosuppressive efficacy.

### 5.3. Opportunities for Further Research

More studies are necessary to better define the role of pre-transplantation weight loss, nutrition counseling, and body composition on lung transplant outcomes, including survival and quality of life [28]. In addition to nutrition counseling, to best maintain muscle mass during intended periods of weight loss, candidates benefit from pulmonary rehabilitation (pre-habilitation) at regular intervals. However, the impact of pre-habilitation on nutrition status remains understudied. Some patients experience unintentional weight loss while awaiting transplant. How this might affect transplant outcomes compared to intentional weight loss is unknown. Lastly, even though nutrition counseling is provided, given the time patients may remain on the waiting list, the optimal frequency of nutrition consultations to best achieve and maintain a healthy nutritional state in patients awaiting transplant is unclear.

## 6. Conclusions

In this short review, we sought to review the use of BMI as a metric of nutrition status in the evaluation of potential lung transplant recipients. Because of its low cost, ease of use, and studies linking outcomes to BMI extremes, BMI ranges are currently utilized in published guidelines as absolute or relative contraindications to lung transplant. However, the use of BMI alone to assess nutrition status may lead to miscalculation of a candidate’s true nutrition status. Body composition analysis through DXA, CHI, lean psoas area, or measured leptin levels can augment BMI through the identification of sarcopenia in overweight/obese patients or higher than anticipated lean muscle mass in underweight patients. Further, repeated measures in patients awaiting transplant can help to determine the success of pre-transplant nutritional interventions.

The role of the dietitian is critical in the multidisciplinary review of potential lung transplant candidates. Dietitians provide diet education and healthy weight loss counseling in overweight and obese patients, with emphasis on slow steady loss in coordination with pulmonary pre-habilitation services to prevent muscle wasting. In underweight patients, dietitians develop a strategy for healthfully gaining weight. They may provide oral nutrition supplements or suggest the initiation of nutrition support (enteral or parenteral) should it be deemed essential. With adequate time, as a result, patients may lose unwanted fat, gain muscle tone, and improve their overall nutrition status and candidacy for transplant. Similar measures can be taken following transplant to improve or maintain nutritional status with the goal of optimizing post-transplant outcomes.

## Figures and Tables

**Table 1:** Summary of studies reviewed.

Author	Year	Study Type	Number of Patients	Nutrition Assessment	Results
Plöchl [8]	1996	Single center, retrospective	51	BMI	BMI in lowest quartile associated with ICU mortality in patients requiring >5-day ICU LOS
Schwebel [9]	2000	Single center, retrospective	78	CHI	Low lean body mass associated with more severe hypoxemia, reduced 6MWT, and higher mortality pre-transplant and longer post-transplant mechanical ventilation and ICU LOS
Madill [10]	2001	Single center, retrospective	251	BMI	Higher risk of post-transplant 90-day mortality in patients with BMI of ≤17 kg/m^2^ or ≥25 kg/m^2^
Kanasky [5]	2002	Single center, retrospective	85	BMI	3X increased risk of post-transplant mortality for obese (BMI > 30 kg/m^2^) patients, but no difference between overweight (BMI 25–29.9 kg/m^2^) and normal weight patients
Singer [11]	2014	Multicenter, retrospective	599	Leptin/DXA	Elevated leptin levels, but not BMI 30–34.9 kg/m^2^, were associated with increased mortality
Weig [12]	2016	Single center, retrospective	103	LPA	Lower LPA associated with longer mechanical ventilation, need for tracheostomy, and ICU LOS

6MWT: 6-min walk test; BMI: body mass index; CHI: creatinine-height index; DXA: dual x-ray absorptiometry; ICU: intensive care unit; LOS: length of stay; LPA: lean psoas area.
